# Everybody's Page

**Published:** 1888-06-30

**Authors:** 


### Everybody's Page.

The sick-rate in our large towns is 36 per cent. in excess the sick-rate in our rural districts.

The young animal is more readily influenced by external conditions than the adult, and the absence of light interferes with its development, even with its existence.

According to the *Mail and Express*, a strong solution of cocaine will stop the irritation of mosquito-bites, and prevent swelling. This discovery is very well, but something to render the human body offensive to mosquitos and sand-flies would be still better.

The kind of food taken by cows alters the milk both in taste and appearance. Madder and saffron will colour it; the odour will be altered by taking plants of the onion tribe, and the taste by such articles as turnips. Milk may acquire medicinal properties by cows feeding on certain plants, and this method of treating diseases has been suggested.

The time when the choice of a life's work has to be made is just the time when there is the greatest risk of consumption supervening, great care should therefore be exercised in choosing. Of course the country or open air is the best, but few can get this. In town life ventilation should be carefully attended to, and open air exercise systematically taken—walking and climbing—and for those who are young and strong, cricket, football, and tennis are of the greatest value.

Some time ago we alluded to an ointment which had been recommended to us, which professed to relieve diseases of bones, by hastening the removal of dead sequestra, and thus obviating the necessity, in many cases, of amputation and serious operations. There was perhaps a tone of flippancy in our remarks upon this wonderful ointment, and we complained that we were asked to recommend to our readers a drug of whose constituents we were ignorant. Our words seem to have grieved the honourable and gallant gentleman who stands sponsor for the ointment, and he has courteously sent us the recipe, which runs as follows: "Half-pint linseed oil, 1 ounce bees' wax, half-pound white resin, ¾ drachm verdigris. Melt; stir till cool. Spread very thin on rag, and place on wound." Any of our readers who care to try the medicament can therefore have it made up from this prescription, and the only duty left to us is to add a warning to the impatient. The gentleman who is endeavouring to bring it before the notice of the public, of whose *bona fides* we entertain not the smallest doubt, makes the following admission: "In the General Infirmary (Derby) I am told that the operation of the ointment is too slow, eighteen months being the shortest time in which a limb is saved."

The change which takes place in air during the act of respiration is very decided. When taken in a dry state, the atmosphere in country places or rural districts contains from 20·96 to 20·98 per cent of oxygen, and only 0·04 per cent. of carbonic acid, and in town districts from 20·87 to 20·90 per cent. of oxygen, and 0·04 per cent. of carbonic acid. The proportion of the latter is therefore very small, and indeed is only 4 parts in 10,000, or 1 part in 2,500 parts. But when the air is breathed out again it contains only about 18·50 per cent. of oxygen, and 4·00 per cent. of carbonic acid, so that whilst the oxygen has diminished by about 3 per cent., the carbonic acid has increased to 100 times the original quantity. Such air must be very different in quality from the ordinary atmosphere, and indeed when it is collected in a jar, and a lighted candle is introduced, the candle is extinguished, and when an animal is placed in it, the animal quickly dies. The air, therefore, wl ich we breathe in is flame-and-life-inspiriting air, but the air we breathe out is flame-and-life-destroying air.

According to the *Medical Press*, a committee appointed by the Academy of Medicine in Belgium, to investigate and report on the effects of public exhibitions of hypnotism, reports that they are hurtful physically and morally, and recommends their prohibition.

In a progressive civilisation such as prevails in this country and throughout the greater part of Europe and America, there is reason to believe that the cranial capacity of the population is, on the whole, increasing rather than diminishing. Owing to the want of early observation, it is difficult to institute comparisons between past and present. An opportunity, however, lately occurred in Paris which was taken advantage of by M. Broca. In digging the foundation of a new building a vault was opened containing a large number of human skeletons, whose surroundings proved them to have lived not later than the twelfth century. M. Broca found the average capacity of 115 of those twelfth-century skulls to be 1,426 cubic centimetres; while another series of skulls—125 in number—taken from a cemetery belonging to the early years of the present century, gave an average of 36 cubic centimetres more. The average Parisian skull would thus seem to have increased considerably in capacity during seven centuries of progressive civilisation.

The duration of hair life is limited, and sooner or later it is shed. Indeed it is stated that the hairs of an infant are completely shed within a year after birth; those on the body and limbs go first, whilst the hairs of the head and the eyelashes follow. This change may be carried on almost imperceptibly, as the place of the falling hairs is taken by a second crop. The process of loss and renewal is very simple. The old hair is detached from the papilla, and soon another hair makes its appearance at the bottom of the same follicle, and grows towards its orifice. The detached hair is thus gradually thrust out and shed. It is not at all uncommon to see the two hairs—the young and the old—projecting from the mouth of the same follicle. The whole process is somewhat similar to the replacement of the milk teeth in the child by the permanent teeth. The second crop of hair which appears is perennial. Each hair has been calculated to remain attached from two to six years. Before it dies, provision is made for its successor, and so this process of shedding and renewal goes on continually.

A German physician practising in the Riviera gives in the *Deutsch. Med. Zeit.* the following particulars, illustrating the difficulty and uncertainty attending the dispensing of foreign prescriptions: A chemist in Genoa told him that about nine-tenths of the prescriptions which were brought to him by foreigners came from abroad. Those from Germany are of course in Latin, "a by no means familiar language to the Italian pharmacist" (!). Besides, as neither a German Pharmacopœia nor a Latin-Italian Dictionary is usually to be found, sad havoc must often be made of the intentions of the prescriber. There is, says the writer referred to, no official Pharmacopœia, and so, even when a prescription is written in Italian, the greatest care has to be taken to indicate exactly what preparations are meant. The Pharmacopœias mostly in use seem to be that of Orosi (1876) and Dorvault's French *Officine*. There are also other Italian Pharmacopœias, as those of Sardinia (1853) and the Papal States (1869), and the old Austro-Italian one. In one chemist's shop in Genoa he found nine Italian Pharmacopœias (most of them imperfect), two English and two French ones. There is no proper control over the pharmacies, and therefore old and useless drugs may be sold; still, he says it is quite possible to obtain as good drugs in the Riviera as elsewhere.

